# Combined Effect of Cadmium and Lead on Durum Wheat

**DOI:** 10.3390/ijms20235891

**Published:** 2019-11-24

**Authors:** Alessio Aprile, Erika Sabella, Enrico Francia, Justyna Milc, Domenico Ronga, Nicola Pecchioni, Erika Ferrari, Andrea Luvisi, Marzia Vergine, Luigi De Bellis

**Affiliations:** 1 Department of Biological and Environmental Sciences and Technologies (DiSTeBA), Salento University, Via Prov. le Lecce-Monteroni, 73100 Lecce, Italy; alessio.aprile@unisalento.it (A.A.); andrea.luvisi@unisalento.it (A.L.); marzia.vergine@unisalento.it (M.V.); luigi.debellis@unisalento.it (L.D.B.); 2 Department of Life Science, University of Modena and Reggio Emilia, via Amendola 2, 42122 Reggio Emilia, Italy; enrico.francia@unimore.it (E.F.); domenico.ronga@unimore.it (D.R.); nicola.pecchioni@unimore.it (N.P.); 3 Department of Chemical and Geological Sciences, University of Modena and Reggio Emilia, via G. Campi 103, 41125 Modena, Italy; erika.ferrari@unimore.it

**Keywords:** cadmium, lead, nicotianamine, mugineic acid, heavy metal, toxic metal, durum wheat

## Abstract

Cadmium (Cd) and lead (Pb) are two toxic heavy metals (HMs) whose presence in soil is generally low. However, industrial and agricultural activities in recent years have significantly raised their levels, causing progressive accumulations in plant edible tissues, and stimulating research in this field. Studies on toxic metals are commonly focused on a single metal, but toxic metals occur simultaneously. The understanding of the mechanisms of interaction between HMs during uptake is important to design agronomic or genetic strategies to limit contamination of crops. To study the single and combined effect of Cd and Pb on durum wheat, a hydroponic experiment was established to examine the accumulation of the two HMs. Moreover, the molecular mechanisms activated in the roots were investigated paying attention to transcription factors (bHLH family), heavy metal transporters and genes involved in the biosynthesis of metal chelators (nicotianamine and mugineic acid). Cd and Pb are accumulated following different molecular strategies by durum wheat plants, even if the two metals interact with each other influencing their respective uptake and translocation. Finally, we demonstrated that some genes (*bHLH 29, YSL2, ZIF1, ZIFL1, ZIFL2, NAS2* and *NAAT*) were induced in the durum wheat roots only in response to Cd.

## 1. Introduction

The distribution of heavy metals (HMs) in soils is variable from one place to another. In some regions, the HM natural background is higher than in other ones, but industrialization and human activities had strongly, in last decades, modified the concentration of many HMs worldwide [1]. A concern with HMs is that they can easily enter the food chain through consumption of vegetables and plant parts. Indeed, the toxicity is due mainly to chronic exposure by eating HM-contaminated foods. Moreover, since low levels of HMs in soil generally do not affect plant growth and development (no visible symptoms), HMs could endanger human health [2] if adequate counteractions are not implemented. Soil metal contamination usually occurs with a combination of different metals. Cadmium (Cd) and lead (Pb) are considered environmental hazards, as they are toxic for humans and other living organisms [3,4] and the Codex Alimentarius (CDX 193-1995, Amended 2019) has set a maximum level of 0.2 mg kg^−1^ for both Cd and Pb in wheat [5].

Cd is an element of group II B in the periodic table and its atomic number is 48, while Pb belongs to group IV A and its atomic number is 82. Cd and Pb can form complexes with other compounds. In particular, Cd could form complexes with ammonia, amines, halide ions and cyanide [6]. In soils, Pb makes complexes with inorganic constituents (e.g., HCO_3_^−^, CO_3_^2−^, SO_4_^2−^ and Cl^−^), or may occur as organic ligands [7].

Although, Cd and Pb are not essential elements, plants are able to adsorb these metals from the soil and store them on different edible organs [8,9]. For these reasons, the control of HM accumulation in plant edible organs is a key point to preserve human health.

Plants respond to HM toxicity activating several physiological and molecular mechanisms. Such responses include immobilization, exclusion, chelation and compartmentalization of the metal ions, and the expression of common stress-related genes such as those involved in ethylene pathway and genes coding for stress proteins [10]. The current approach in risk-evaluation of HMs accumulation in plants is almost always based on the effects of single contaminants [11], but the combined effect of HMs is still low investigated.

Durum wheat is a staple food used to produce pasta (mainly in Europe and America), couscous and freekeh (Africa), bulgur (Asia) and bread (South Italy); minor crop if compared to bread wheat, it is mainly cultivated in Europe (Italy and France), America (Canada, USA, Mexico and Argentina), North Africa and Asia (Ukraine, Russia, Kazakhstan Indi and, China). These regions have different climate conditions and soil types with variable levels of toxic metals. In recent years, many authors have reported information about metal accumulation in durum wheat tissues, such as Cd [12,13], copper [14], arsenic [15], nickel [16], Pb, copper and chromium [17]. However, little is known about the combined effect of these metals on plant development, metal compartmentalization in plant tissues and molecular responses. Recently Shafiq et al. [18] reported how the expression of Heavy Metal ATPase 2 and ATP-Binding Cassette and promoter methylation could have a central role in Cd, Pb and zinc accumulation.

The aim of this work was to investigate the uptake and translocation of Cd and Pb, their interaction in root and leaf tissues and the molecular mechanisms activated by the aboveground presence of one or both metals during the growth of durum wheat plants. We employed two near-isogenic lines (NILs) [19] with an opposite behavior concerning Cd accumulation in leaves, low Cd (L-Cd NIL) and high Cd (H-Cd NIL), respectively. Since Cd accumulation is strongly affected by specific genomic regions [20], we should observe similar accumulation trends in these plants if the same genome regions are involved in Pb accumulation. Moreover, we added to the experiment design two commercial cultivars already characterized for their accumulation and responses to Cd [13] and, with this experiment, we evaluated how the contemporaneous presence of the Cd and Pb can affect each other.

## 2. Results

### 2.1. Levels of Cd and Pb in Root and Leaf of Wheat Plants

**Figure 1:**
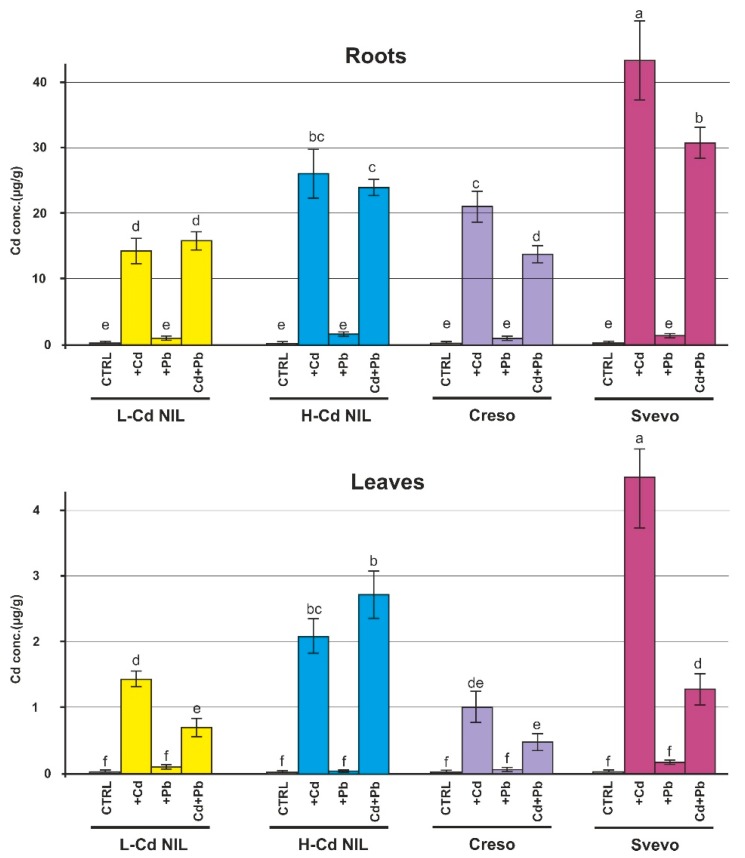
Cadmium (Cd) concentration in roots and leaves of low cadmium near-isogenic line (L-Cd NIL), high cadmium near-isogenic line (H-Cd NIL), Creso and Svevo. Cd concentrations in durum wheat genotypes grown in standard hydroponic solution in the presence of Cd 0.5 μM, lead (Pb) 2.0 μM or in the presence of both heavy metals (HMs) (Cd 0.5 µM plus Pb 2.0 µM). Roots and leaves were collected 42 days after germination (at the tillering stage). Cd concentration was quantified by inductively coupled plasma mass spectrometer (ICP-MS). Statistical analysis was performed through ANOVA (*p*-value < 0.05, *n* = 3) followed by Tukey-HSD post hoc test. Different letters correspond to statistically different means.

**Figure 2:**
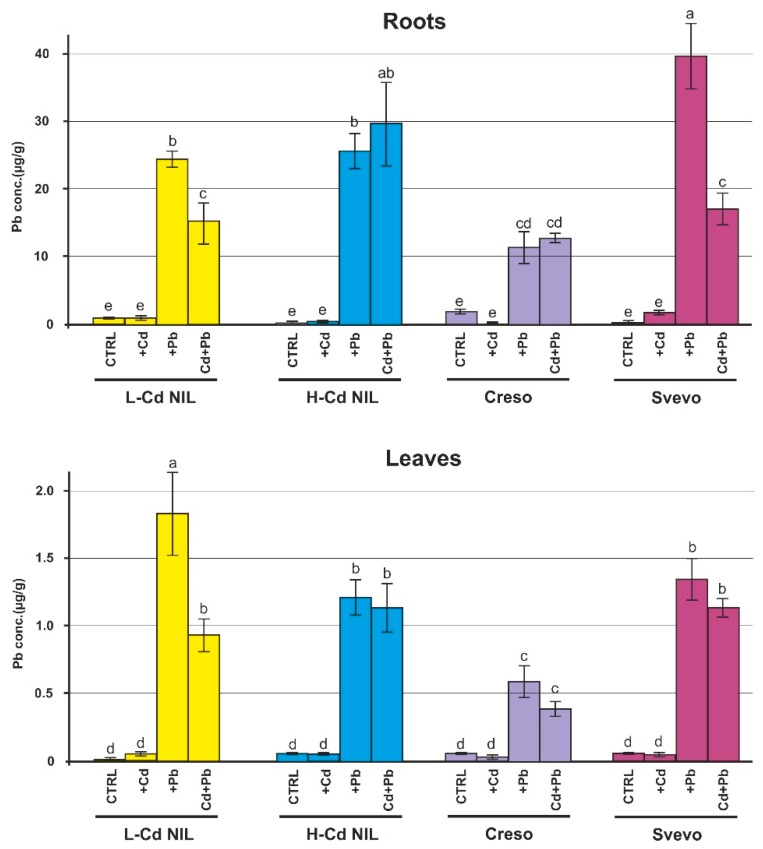
Pb concentration in roots and leaves of L-Cd NIL, H-Cd NIL, Creso and Svevo. Pb concentrations in durum wheat genotypes grown in standard hydroponic solution in the presence of Cd 0.5 μM, Pb 2.0 µM or in the presence of both HMs (Cd 0.5 µM plus Pb 2.0 µM). Roots and leaves were collected 42 days after germination (at the tillering stage). Pb concentration was quantified by ICP-MS. Statistical analysis was performed through ANOVA (*p*-value < 0.05, *n* = 3) followed by Tukey-HSD post hoc test. Different letters correspond to statistically different means.

Samples were collected 42 days after germination, at the tillering stage, from plants grown in hydroponic solutions with the addition of Cd or Pb or with both metals at the concentrations of 0.5 and 2.0 μM, respectively. Figure 1 and Figure 2 show the concentration of the two HMs in roots and leaves of the wheat plants; in roots, Cd and Pb concentrations range from 10 to 40 μg/g dry weight. The presence of a higher level of Cd and Pb in roots is evident (Figure 1 and Figure 2): approximately, the concentration in roots is ten-times higher than in leaves. The Cd concentration in leaves expressed as µg/g dry weight is also twice as high as that of Pb and about four times if expressed as molarity because of the different atomic weight of the two metals.

L-Cd NIL, the near isogenic line characterized by the ability to accumulate a low level of Cd in leaves, collected, as expected, a low level of Cd in leaves compared to H-Cd NIL and to Svevo (Figure 1), whereas it showed the presence of a high level of Pb in leaves in comparison with all other genotypes (Figure 2), suggesting the presence of different molecular mechanism for the transport of the two metals. In L-Cd NIL the co-presence of Pb reduced the Cd accumulation in L-Cd NIL leaves (Figure 1).

Despite H-Cd NIL theoretically shares about the 95% of the genome with L-Cd NIL, it accumulates a high level of Cd in leaves [17], as confirmed in Figure 1. The co-presence of Pb did not alter significantly the concentration of Cd, which remained at comparable levels in both roots and leaves. Instead, L-Cd NIL and H-Cd NIL showed a similar behavior concerning the uptake and translocation of Pb and the co-presence of Cd altered considerably the Pb accumulation only in L-Cd NIL leaves (Figure 2).

The accumulation of Cd in Creso was influenced by the co-presence of Pb in hydroponic solution: when Creso was treated with both metals, the Cd concentrations in roots and leaves were slightly lower indicating a negative effect of Pb on Cd uptake/translocation.

Svevo, compared to the other genotypes, resulted more sensitive to the occurrence at the same time of two metals in the hydroponic solution. Indeed, in Svevo the addition of a second metal strongly reduced the accumulation of the other one. This behavior was observed in roots (both Cd and Pb) and in leaves (only Cd was significantly reduced after the application of Pb).

**Figure 3:**
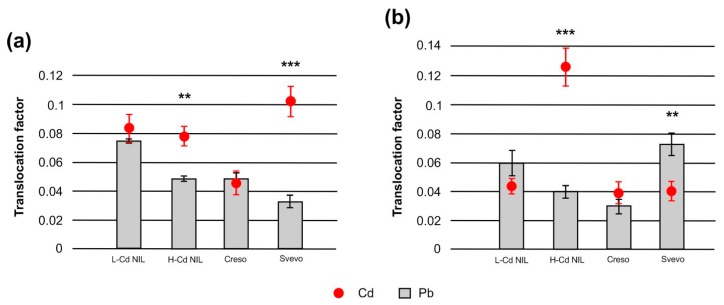
Translocation factor of Cd and Pb from root to shoot of wheat plants, (**a**) after the single treatments with Cd and Pb and (**b**) after the combined treatment with the two HMs. The significant differences, between the single heavy metal treatments, were highlighted according to Student’s *t*-test (* *p* < 0.05; ** *p* < 0.01; *** *p* < 0.001).

An important parameter to study HMs uptake and translocation in plants is the translocation factor (Figure 3); it is the ratio of the metal concentration in other plant tissues in relation to roots [3]. Figure 3 indicates that the translocation factors from roots to leaves for Cd and Pb were clearly lower than 1.0 (ranging around 0.1 or less as it results from the values on the axes) indicating a robust limitation in Cd and Pb transport in durum wheat and an immobilization in the root cells; this strategy is widely used by plants to protect the photosynthetic tissues from damages caused by the HMs [21]. Anyway, translocation factor values showed that in the genotypes L-Cd NIL and Creso, Cd and Pb were translocated from root to shoot with a similar ratio; conversely, in Svevo and H-Cd NIL, Cd was translocated from root to shoot more efficiently than Pb compared with the other genotypes (Figure 3a). This is an expected result since Svevo and H-Cd NIL are well-known genotypes with high grain-Cd accumulation [13,17]. During the combined treatment with Cd and Pb, the genotype H-Cd NIL kept higher translocation factor values for Cd (Figure 3b), while in Svevo, the translocation factor of Pb became significantly higher than the translocation factors of Cd (Figure 3b). This is explainable since in Svevo the HMs combined treatment affected Cd accumulation more at leaves level than in roots (Figure 1) causing a decrease in translocation factor values. In contrast, the combined treatment impacted Pb accumulation at root level more significantly than in leaves (Figure 2), determining an increase in translocation factors values.

Such data may represent a first evidence of the existence of different response mechanisms to Cd and Pb.

### 2.2. Gene Expression in Response to Cd and Pb

The differences in Cd and Pb accumulation observed in the durum wheat genotypes may be induced by differential expression of gene categories involved in metal ion response and transport. The role of these genes has already been investigated in tissues of plants treated with Cd [22]. To study the molecular response to Cd, Pb and their combined effect, we considered the level of expression of genes already known to be involved in HMs responses. We focused the attention on transcription factors (bHLH and WRKY family), eight metal ion transporters and genes coding for the enzyme responsible of the synthesis of nicotianamine and mugineic acid, that are two typical metal chelators of graminaceous plants [23]. The expression levels of these genes were analyzed both in roots and leaves, but we observed no expression or no differential expression in durum wheat leaves, suggesting a tissue-specific regulation/expression. Below, only the root transcription data were reported.

#### 2.2.1. Expression of the Transcription Factors Basic Helix-Loop-Helix (bHLH) and WRKY33

**Figure 4:**
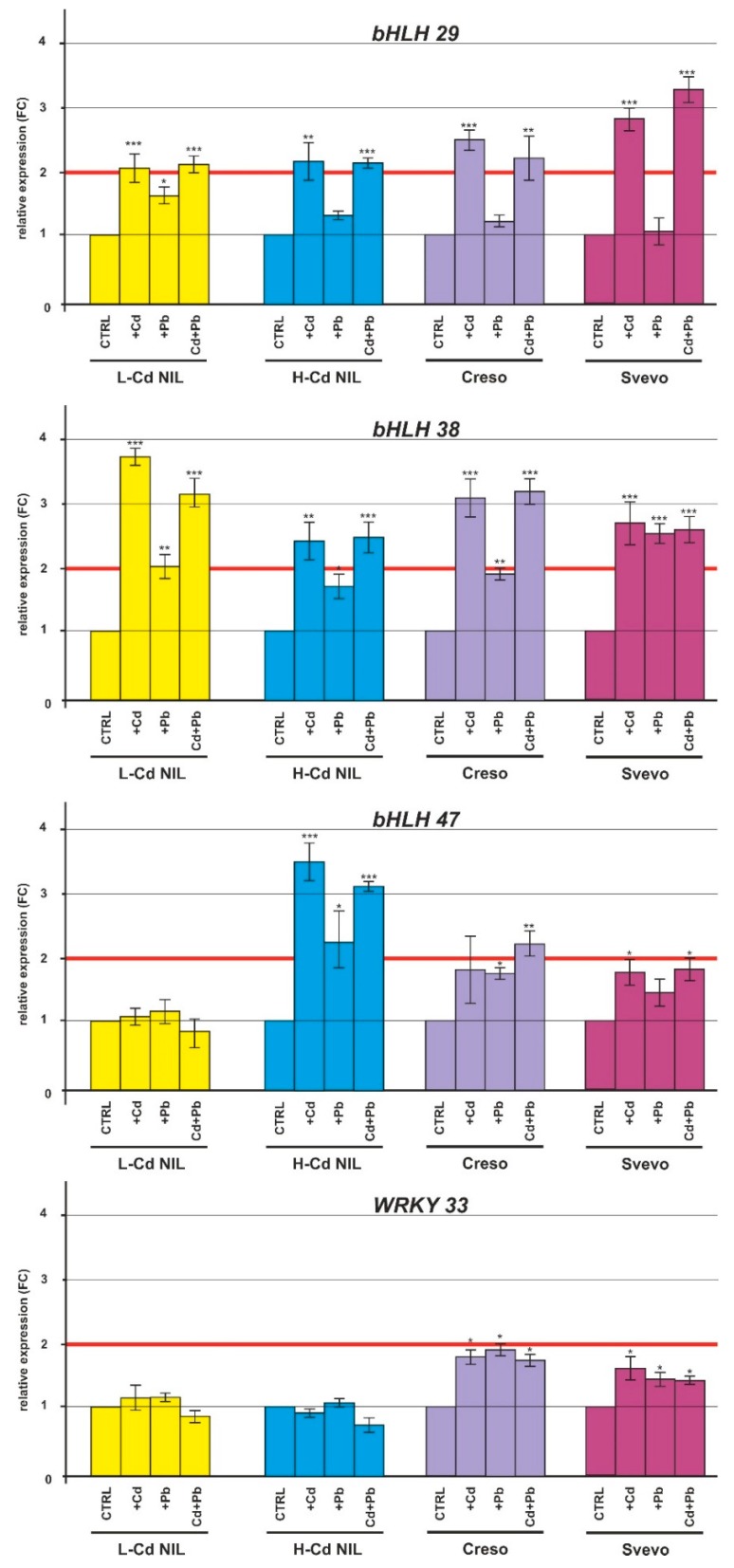
Relative expression as fold change (FC) of the *bHLH29/FIT*, *bHLH38/ORG2*, *bHLH47/PYE* and *WRKY33* genes in root tissues of the durum wheat genotypes (L-Cd NIL, H-Cd NIL, Creso and Svevo) grown in the presence of Cd 0.5 μM, Pb 2.0 µM or in the presence of both HMs (Cd 0.5 µM plus Pb 2.0 µM). Error bars indicate standard deviation of the mean of three technical replicates resulting from a bulk of three biological replicates. ANOVA results were reported basing on their statistical significance. * *p* < 0.05, ** *p* < 0.01, *** *p* < 0.001.

As shown in Figure 4, the expression of *bHLH29/FIT* and *bHLH38/ORG2* was clearly up-regulated in the roots of the four analyzed genotypes when treated with Cd and with Cd plus Pb; on the contrary, *bHLH47/PYE* (Figure 4) was only up-regulated in the genotype H-Cd NIL both after a treatment with Cd or Pb and following the combined stress determined by Cd plus Pb. *WRKY33*, member of the WRKY transcription factors family, did not show a significant modulation in roots of the four genotypes during treatments with the HMs (Figure 4).

#### 2.2.2. Expression of HMs Transporters

**Figure 5:**
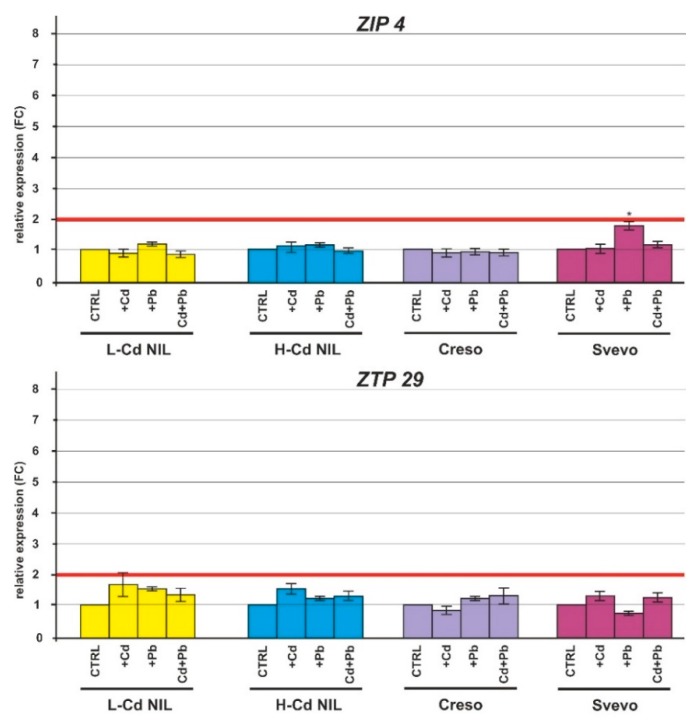

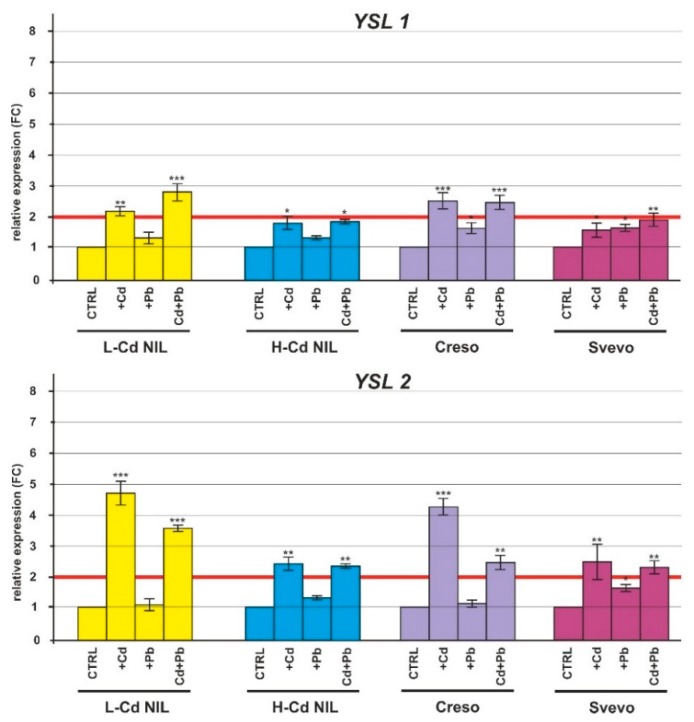
Relative expression (FC) of the *ZIP4*, *ZTP29*, *YSL1* and *YSL2* genes in root tissues of the durum wheat genotypes (L-Cd NIL, H-Cd NIL, Creso and Svevo) grown in the presence of Cd 0.5 μM, Pb 2.0 µM or in the presence of both HMs (Cd 0.5 µM plus Pb 2.0 µM). Error bars indicate standard deviation of the mean of three technical replicates derived from a bulk of three biological replicates. ANOVA results were reported basing on their statistical significance. * *p* < 0.05, ** *p* < 0.01, *** *p* < 0.001.

**Figure 6:**
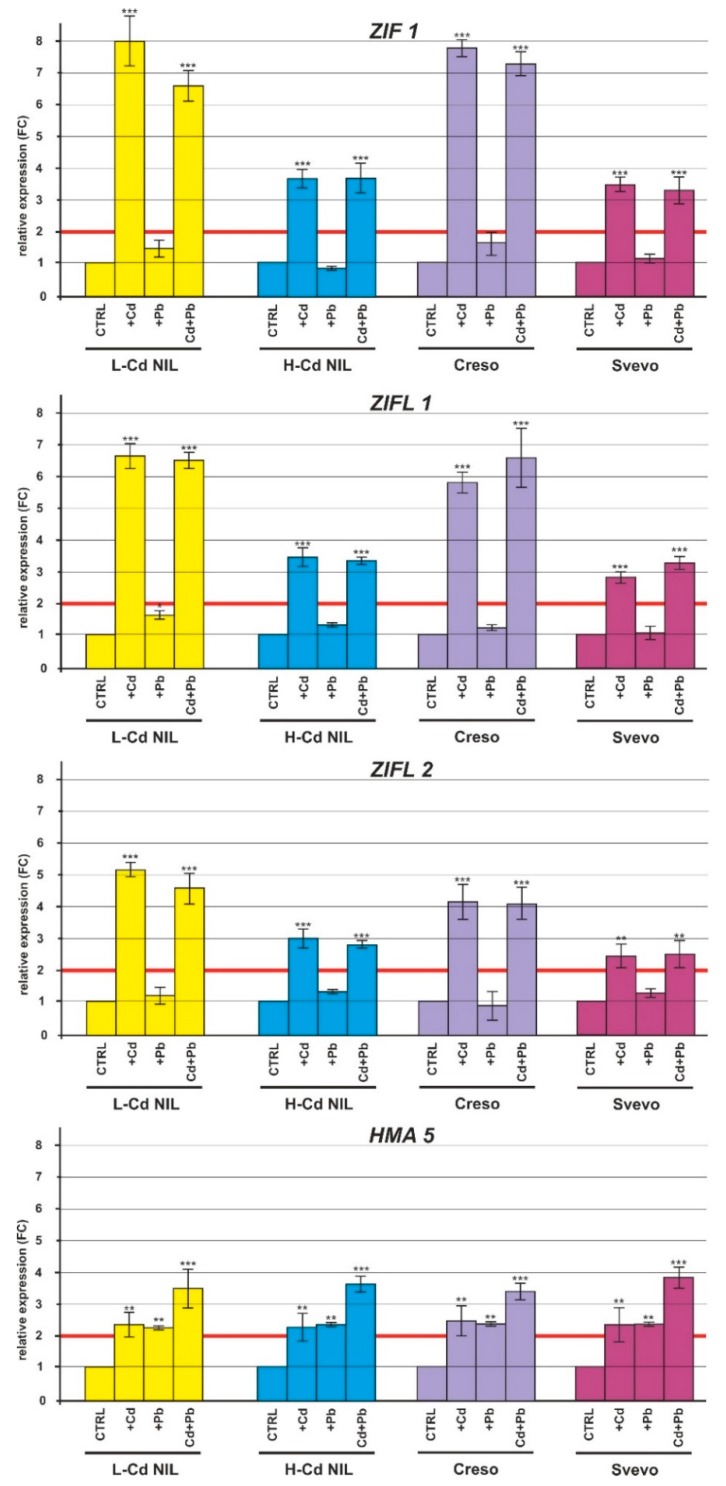
Relative expression (FC) of the *ZIF1*, *ZIFL1*, *ZIFL2* and *HMA5* genes in root tissues of the durum wheat genotypes (L-Cd NIL, H-Cd NIL, Creso and Svevo) grown in the presence of Cd 0.5 μM, Pb 2.0 µM or in the presence of both HMs (Cd 0.5 µM plus Pb 2.0 µM). Error bars indicate standard deviation of the mean of three technical replicates derived from a bulk of three biological replicates. ANOVA results were reported basing on their statistical significance. * *p* < 0.05, ** *p* < 0.01, *** *p* < 0.001.

Plants treated with HMs showed no significant change in transcripts of the genes coding for the HM transporters ZIP4 and ZTP29 in root tissues (Figure 5). *YSL1* was slightly up-regulated in the roots of the L-Cd NIL and Creso when treated with Cd or with Cd plus Pb (Figure 5); conversely, expression level of *YSL2* was strongly up-regulated in roots of L-Cd NIL and Creso when treated with Cd. The presence of both Cd and Pb resulted in a significant increase of the transcript levels in roots of L-Cd NIL; in roots of the cultivar Creso, the combined stress induced a slight upregulation (Figure 5). A slight up-regulation was observed also in H-Cd NIL and Svevo roots treated with Cd and in the combined treatment Cd plus Pb (Figure 5). The vacuolar zinc transporter genes *ZIF* and *ZIF*-like genes (*ZIFL1* and *ZIFL2*) were strongly up-regulated in the roots of the four durum wheat genotypes treated with Cd and Cd plus Pb (Figure 6). The transcript levels were significantly higher in L-Cd NIL and Creso (Figure 6). Finally, the plasma membrane-localized transporter *HMA5* was significantly up-regulated in roots when treated both with one HM, or with the combined Cd plus Pb (Figure 6).

#### 2.2.3. Expression of the Nicotianamine Synthase Genes (NAS) and Nicotianamine Aminotransferase (NAAT)

**Figure 7:**
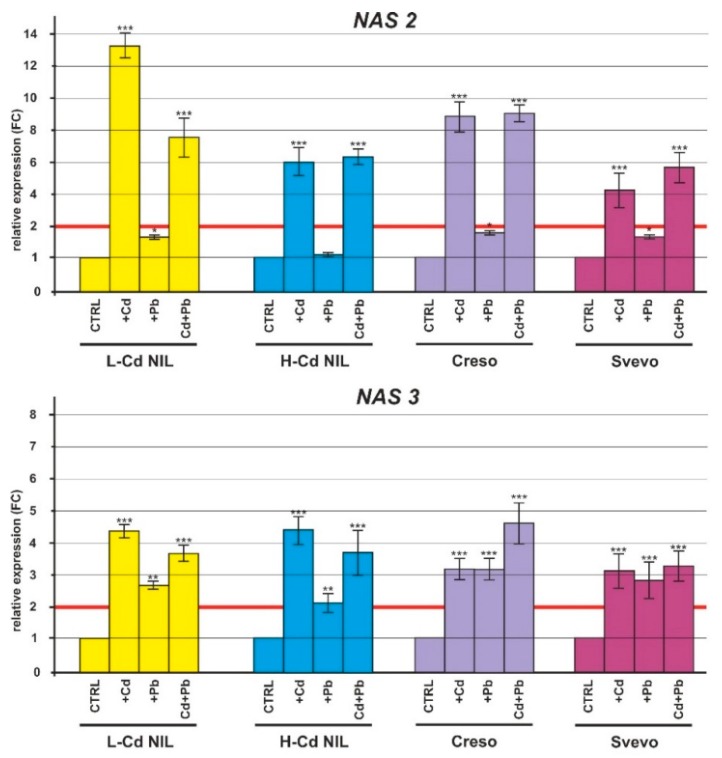

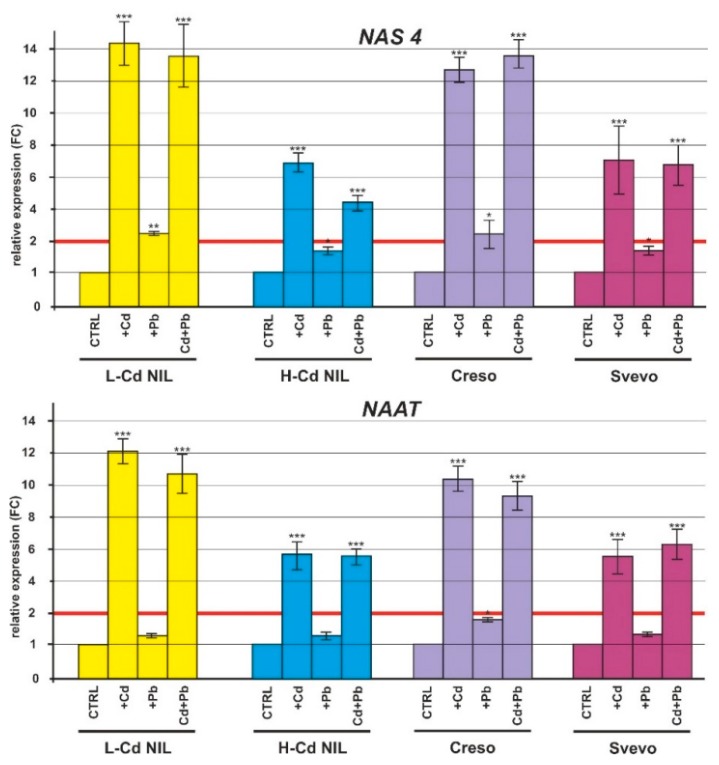
Relative expression (FC) of the nicotianamine synthase genes (*NAS2*, *NAS3*, *NAS4*) and nicotianamine aminotransferase gene (*NAAT*) in root tissues of the durum wheat genotypes (L-Cd, H-Cd, Creso and Svevo) grown in the presence of Cd 0.5 μM, Pb 2.0 µM or in the presence of both HMs (Cd 0.5 µM plus Pb 2.0 µM). Error bars indicate standard deviation of the mean of three technical replicates derived from a bulk of three biological replicates. ANOVA results were reported basing on their statistical significance. * *p* < 0.05, ** *p* < 0.01, *** *p* < 0.001.

In the current study, the transcripts levels of the nicotianamine synthase genes (*NAS2*, *NAS3* and *NAS4*) confirmed their up-regulation in roots of plants treated with Cd (Figure 7) as observed by other authors [19]. Interestingly, a significant increase of the transcript levels for the genes *NAS2*, *NAS3* and *NAS4* were also found in roots of plants treated with Cd plus Pb (Figure 7). Moreover, the gene *NAS3* resulted modulated in response to Pb treatment too, with a minor induction in roots (Figure 7). The transamination of nicotianamine produces mugineic acid and the enzyme that catalyzes the synthesis is called nicotianamine aminotransferase (NAAT); the relative gene is expressed at highest levels in roots of L-Cd NIL and Creso grown in the presence of Cd and Cd plus Pb; a significant induction was also found in H-Cd NIL and Svevo exposed to Cd and Cd plus Pb (Figure 7).

## 3. Discussion

The data support the hypothesis that the effects produced by combinations of HMs could have a different impact on both accumulation and gene expression in comparison to the individual effects of each metal. The analyzed durum wheat genotypes showed different distribution of Cd and Pb in roots and leaves when treated with the two HMs in comparison with a treatment with only one metal. In general, the four genotypes showed a reduced accumulation of both Cd and Pb in the combined treatment (Figure 1 and Figure 2). Several studies described that the presence of one metal influenced the uptake of another metal [24,25]. Xie et al. [25] investigated the effects of combined HMs toxicity on two rice genotypes differing in Cd accumulation; they found that the application of Pb, Cd, chromium (Cr) and copper (Cu) significantly affected grain Cd accumulations. In rice, Zeng et al. [26] also reported a significant Cd and Pb reduction in grains when exposed to both metals. Other studies were carried out on cucumber (*Cucumis sativus* that could retain greater amount of metals in the roots due to its root morphology) [27] to assess HMs toxicity in soils contaminated by Cu, Cd and Pb separately and in combinations; according to their results, bioaccumulation of one metal was influenced by the presence of other metals and, in general, the HMs accumulation patterns reflected antagonistic and/or synergistic plant’s responses. In the binary combination of Cd and Pb, they found a synergistic response with a reduction of toxicity effect [28]. Cd/Pb synergisms have been previously reported by Zaray et al. [29]. On *Lemna minor*, a metal pollution sensitive plant, the combined toxicity of Pb and Cd was found to be less effective when compared to the toxicity of the individual treatment [30].

The data about Cd and Pb accumulation in leaves highlighted different behaviors among genotypes in relation to the two toxic metals. Svevo and H-Cd NIL are good accumulators of both Cd and Pb in leaves. On the contrary Creso accumulates lower level of Cd and Pb if compared to the other genotypes. The L-Cd NIL has a contrasting behavior: accumulates low level of Cd and high level of Pb. It is interesting to note that the expression levels of the tested genes in Svevo and H-Cd NIL are generally less up-regulated or not-regulated at all, suggesting that these genes are involved in some molecular mechanisms to stuck Cd and Pb at root level (e.g., genes involved in vacuole compartmentalization of toxic metals).

These differences (both in accumulation and distribution of HMs) could be, in part, due to differential expression of genes involved in HM uptake, cellular sequestration and translocation from root to shoot. Since among the HMs, Cd is accumulated in the grain of durum wheat to levels exceeding the Codex Alimentarius Commission standards [5] Cd uptake, cellular sequestration and translocation have been thoroughly studied [31,32]; some of the genes, with a key role in these physiological steps in durum wheat plants during Cd treatment [22], were investigated during the combined exposure to Cd and Pb.

Cd can enter root cells as Cd^2+^ through ZIP (zinc regulated transporter/iron regulated transporter-like protein) transporters or as Cd-chelates through YSL (Yellow-Stripe 1-Like) proteins [33]. Some investigations have proven that the ZIP family transporters participate in Cd absorption and accumulation in plants [34]; in our work, the selected gene *ZIP4* was not significantly regulated (Figure 5) in response to Cd treatment. Yamaguchi et al. [35] also found that genes coding for ZIP transporters did not change their expression patterns in *Solanum torvum* roots during treatment with low Cd concentrations and they postulated that the absence of changes in these metal transporters may explain why the mild Cd exposure did not induce serious competitive inhibitory effects on metal ion homeostasis in roots. The same effect due to the exposure to a low HM concentration could explain the unchanged expression of the gene *ZTP29* (Figure 5). This gene, coding for a zinc transporter with homology to the *Arabidopsis ZTP29*, is localized into the endoplasmic reticulum and it is thought to play a role in the unfolded protein response [36], Liu et al. [37] found that Cd up-regulated *ZTP29* in roots of *Cosmos bipinnatus* Cav. When they are under 40 μmol/L Cd stress (80 times more concentrated than the concentration used in this work).

Instead, the genes coding for the YSL (Yellow-Stripe 1-Like) proteins responsible for the transport of the Cd-chelates resulted up-regulated in Cd and Cd plus Pb treatments with higher FC values in the genotypes L-Cd NIL and Creso in which also *YSL2* was vigorously activated (Figure 5). This upregulation in response to Cd treatment is in accordance with RNA sequencing data reported in Aprile et al. [22]. The YSL family of transporters represents a candidate for the transport of nicotianamine (NA)–metal chelates across plant cell membranes [38]; several members of the YSL family are localized to the plasma membrane and function as transporters of metals that are bound to the metal chelator nicotianamine or the related set of mugineic acid family chelators known as phytosiderophores [39]. Other YSL members are localized to the vacuole membranes and to the internal membranes and may play a role in detoxification by HM sequestration in the vacuole [40]; this compartmentalization mechanism could contribute with the characteristic trait of low Cd-accumulation in durum wheat grain [13]. The two genotypes (L-Cd NIL and Creso) with lower Cd and Pb accumulation in leaves, had also the higher level of expression of the gene *YSL2*, suggesting a possible regulatory role in Cd and Pb compartmentalization in roots. The treatment with Pb did not affect the expression level of the *YSL* transporters while the Cd-Pb combined treatment affects the *YSL2* expression in the L-Cd NIL and Creso by reducing the amount of mRNA in comparison with the expression level in the single Cd treatment; the exposure to the combined HMs can activate simultaneously several nonspecific defense systems and it is reported that the interactions among HMs affect their uptake and accumulation in plants [25,26,31,41]. Other NA vacuolar transporter genes are the *ZIF* and *ZIF*-like genes, their expression levels were up-regulated by Cd and Cd plus Pb treatments in all the analyzed genotypes while the Pb treatment did not change expression pattern (Figure 6); so it is reasonable to assume that the Cd-nicotinamine chelates could enter into the vacuoles through the ZIF and ZIFL.

HMs are loaded from the symplast into the xylem by heavy metal P_1B_-ATPases, known as heavy metal ATPases (HMAs) that play an important role in metal transport in plants [20,33].

In our experiment, the gene coding for the transporter HMA5 was upregulated both in Cd and Pb single treatment and the combined treatment increased the amount of mRNA if compared with the HM single treatment as if there was a cumulative effect (Figure 6). Functional studies on the HMAs have shown that these transporters can be divided into two subgroups based on their metal-substrate specificity: a copper (Cu)/silver (Ag) group and a zinc (Zn)/cobalt (Co)/Cd/Pb group [42]; this indication is in accordance with our data since *HMA5* was induced both by Cd and Pb. Besides genes coding for HMs transporters, previous studies have characterized several transcription factors (TFs) involved in Cd response: ERF, WRKY and bHLH TF families [43]. Obtained gene expression patterns supported the involvement of these genes in the response to Cd stress since *bHLH29/FIT*, *bHLH38/ORG2* and *bHLH47/PYE* were significantly induced only in response to Cd treatment (Figure 4). A significant up-regulation in response to Pb treatment was recorded exclusively in the genotype Svevo for the gene *bHLH38/ORG2* (Figure 4). For the TF *WRKY33* not significant induction was observed (Figure 4). Since many transcription factors are transiently regulated by stresses/treatments, the long-term exposure to Cd and Pb and the sample collection at 42 days after germination were not suitable to observe a transcription variation. In *Arabidopsis thaliana* treated with Cd, real-time PCR analyses demonstrated that the *WRKY13* transcript was rapidly induced by Cd stress, reaching the maximum level after 1 h of Cd treatment and gradually decreasing, thereafter [44]. On the other hand, Long et al. [45] described how the co-overexpression of *FIT* and *ORG2* enhanced the expression of nicotianamine synthase 1 (*NAS1*) and *NAS2*, resulting in the accumulation of nicotianamine, a crucial chelator for Fe transportation and homeostasis.

Figure 7 showed a strong induction for the genes *NAS2*, *NAS3* and *NAS4* in response to Cd treatment while no gene expression modulation was recorded in response to Pb treatment. These data suggested that the molecular mechanisms regulated by the bHLH TFs are well conserved between *Arabidopsis* and durum wheat and also gave evidence of a detoxification mechanism specific for Cd stress since Pb treatment did not activate the synthesis of nicotianamine. According to Pal and Ray [46], phytochelatins synthesis is influenced by the metal ion treatment and a number of phytochelatins variants have been found among plant species. Nicotianamine (NA) is a non-proteogenic amino acid chelator having more than one binding centers, which confer high affinity for Fe, but also for other metals such as Zn, Cu, Mn, Ni and Cd [46]. Another chelator, involved in iron uptake in graminaceous plant species, is the mugineic acid [47]. In the mucigenic acid biosynthetic pathway, nicotianamine aminotransferase (NAAT) is implicated in the formation of 2′-deoxymugineic acid (DMA) from nicotianamine [48]. With our expression data, we described that *NAAT* was highly expressed in root tissues of all the analyzed genotypes treated with Cd (Figure 7) indicating a critical role for the mucigenic acid in response to Cd.

## 4. Materials and Methods

### 4.1. Genetic Materials

To identify the effects of the combined stress of Cd and Pb in durum wheat (*Triticum turgidum* L. subsp. *Durum*), a pot experiment was conducted in a growth chamber. Two low grain Cd accumulation (L-Cd NIL and Creso) and two high grain Cd accumulation (H-Cd NIL and Svevo) were analyzed. Creso and Svevo accession numbers are, respectively, K-53049 and RICP-01C0107074, and all pedigree information is browsable at CIMMYT database (http://www.wheatpedigree.net). L-Cd NIL and H-Cd NIL are two near-isogenic lines (NILs) of durum wheat (*Triticum turgidum* L. subsp. *durum*) that differ in grain Cd accumulation TL 8982-H (H-Cd NIL) and TL 8982_L (L-Cd NIL) [17].

### 4.2. Experimental Design

Cd and Pb management to the four genotypes was set up by hydroponic system. After external sterilization, seeds were germinated in Petri dishes with moist filter paper, in the dark at 8 °C. After germination (6–7 days), plantlets were located into 0.4 L plastic pots (7 cm × 7 cm × 8 cm) filled with perlite, moistened with tap water, and immediately transferred to the 10 L polyethylene tanks of the hydroponic system, as described by Harris and Taylor [49] with little modifications. In each pot three seedlings were lodged in and for each treatment, three different pots were considered for three biological replicates. The positions of the pots in the growth chamber were completely randomized and changed weekly with a new randomization. Plants were grown in the growth chamber under long days, 16 h light/8 h night, 21 °C/16 °C. The hydroponic solution was given with systematic pauses, irrigating for 15 min every 2 h during the all day; while during the night no fertigation. In this way, the perlite substrate was constantly dampened with hydroponic solution, avoiding stagnation. The nutrient solution was prepared using reverse osmosis (RO) water (<30 μS cm^−1^) and contained: 0.3 mM NH_4_NO_3_, 0.25 mM KNO_3_, 0.1 mM K_2_SO_4_, 50 μM KCl, 1.0 mM Ca(NO_3_)_2_, 0.3 mM Mg(NO_3_)_2_, 100 μM Fe(NO_3_)_3_, 1.0 μM MnSO_4_, 10.0 μM H_3_BO_3_, 10.0 μM ZnSO_4_, 0.2 μM Na_2_MoO_4_, 2.0 μM CuSO_4_, 2.0 μM Cu(NO_3_)_2_, 0.1 mM K_2_HPO_4_, 138.6 μM *N*-(2-hydroxyethyl) ethylenediaminetriacetic acid (HEDTA), and 2 mM 2-(*N*-Morpholino) ethanesulfonic acid hydrate. After preparation of solution, pH was adjusted by 1.42 mM KOH.

The pH of the nutrient solution and its electrical conductivity (EC) were constantly monitored every 2 days, EC was used to estimate water depletion by keeping EC in the main tank between 550 and 600 µS cm^−1^, and nitric acid (1% *v*/*v*) was used to adjust pH between 5.5 and 6.0, when needed.

In cultivated fields the presence of Cd and Pb is usually not toxic for the crops. In this experiment plants were treated by adding to the nutrient solution 0.5 μM CdCl_2_, or 2.0 μM Pb(NO_3_)_2_, or even both in the case of the double metal stress. These concentrations do not cause a significant toxic effect on root and leaf biomass as reported respectively by Harris and Taylor [49] and Sun et al. [50], while Cd 0.5 μM has been employed in previous works of the University of Salento [12,22].

The control plants were cultivated without Cd or Pb in the same hydroponic solution. Hydroponic solution was constantly aerated. One plant for each pot (three for each treatment) was sampled 42 days after germination, at tillering stage (roots and leaves). Roots were easily extracted from perlite substrate and washed manually to remove the perlite beads adherent to roots, and possible excess of hydroponic solution. Leaf samples were washed immediately on harvest in RO water for 30 s, while root samples were triple rinsed (RO water, 1 min; 1 mM CaCl_2_, 5 min; RO water, 1 min), and blotted dry. Samples for quantitative RT-PCR were frozen in liquid nitrogen and then stored at −80 °C.

### 4.3. Inductively Coupled Plasma Mass Spectrometry (ICP-MS) Analysis

Measurements of Cd and Pb uptake in roots were performed by using X SeriesII inductively coupled plasma mass spectrometer (ICP-MS; Thermo Fisher Scientific, Waltham, MA, USA) equipped with Peltier cooled (3 °C) spray chamber. Samples were introduced by the autosampler CETAC ASX 520 into the nebulizer, and the positively charged ions were then produced by a high-temperature, inductively coupled plasma. The ions passed through a sampling cone interface into a high-performance quadrupole mass spectrometer that is computer-controlled to carry out multi-element analysis. Data were analyzed by PlasmaLab software. The instrument was tuned daily with an ICP-MS tuning solution. Yttrium in HNO_3_ 4% (100 ppb) was used as internal standard. Cd and Pb standards ranging from 0.2 to 100 ppb were freshly prepared before each analysis and used to build calibration curve. Each sample was analyzed at least in three independent measurements and each experiment comprised three repetitions. Results are given as mean value ± standard deviation.

Each sample was mineralized by a microwave-assisted procedure performed with an ultraWAWE microwawe digestion system (Milestone Inc., Shelton, CT, USA), as recently reported by Durante et al. [51]. The sample (~300 mg) was accurately weighed in the microwave-quartz vessels before adding 2 mL of ultrapure HNO_3_ 65% *w*/*w* and 4 mL of Milli-Q water. At the end of the digestion process, an almost colorless, pale yellow sample was obtained. The solution was then diluted up to 10 g with Milli-Q water in test tubes. Between each mineralization cycle, a washing cycle was carried out.

Ultrapure HNO_3_ 65% *w*/*w* was obtained from analytical grade nitric acid (Carlo Erba, Milan, Italy) after sub-boiling distillation performed with a sub-boiler SAVILLEX DST 1000 (Savillex Corp. Eden Prairie, MN, USA) apparatus.

### 4.4. Total RNA Isolation, cDNA Synthesis and qPCR Analysis of Gene Expression

To evaluate the response of durum wheat plants to Cd and Pb treatments, we carried out a transcriptomic analysis in a small, but well-defined, group of genes [22]. Total RNA was extracted from root and leaf tissues using TRIZOL reagent according to the method published by Marè et al. [52]. To assess RNA quality and quantity, several dilutions of each sample were analyzed using the Agilent RNA 6000 nano Kit and Agilent Bioanalyzer 2100. cDNA synthesis was performed using TaqMan^®^ Reverse Transcription Reagents (Applied Biosystems, Foster City, CA, USA). qPCR was performed with the Power SYBR Green RT-PCR Master mix (Applied Biosystems, Foster City, CA, USA) according to the manufacturer’s instructions. To calculate the relative expression levels between a reference sample and the related treatments, the fold change (FC) formula was used:

FC = 2^^−ΔΔCT^,

where ΔΔCT = (CT_targetgene_ − CT_referencegene_) _treatedsample_ − (CT_targetgene_ − CT_referencegene_)_controlsample_.

Sequences related to genes coding for transcription factors (*bHLH29*, *bHLH38*, *bHLH47* and *WRKY33*), membrane transporters (*HMA5*, *YSL1*, *YSL2*, *ZIF1*, *ZIFL1*, *ZIFL2*, *ZIP4* and *ZTP29*) and genes involved in the metal chelator pathway (*NAS2*, *NAS3*, *NAS4* and *NAAT*) were downloaded from the site https://www.ebi.ac.uk/arrayexpress, at European Bioinformatics Institute (EMBL-EBI). The accession code is E-MTAB-7266. Then, the sequences were compared to the NCBI database using the BLAST algorithm and the most similar sequences were used to design the relative real-time PCR primers (Primer Express™ Software v3.0, Applied Biosystems, Foster City, CA, USA) Supplementary Table S1. TheNADH ubiquinone reductase gene was used as the reference gene to normalize the expression levels of the target genes.

### 4.5. Statistical Analyses

Means of quantitative data related to Cd and Pb concentrations were determined for each tissue (root and leaf) and were subjected to two-way ANOVA analysis (genotype X treatment), followed by Tukey-HSD (honestly significant difference) post hoc test (*p* < 0.05). Translocation factor data were subjected to two-way ANOVA (genotype X treatment). A *t*-test was employed to find statistical differences between Cd and Pb translocation factors on each genotype. A one-way ANOVA and *t*-test were applied to expression gene data.

Analyses were achieved using R version 3.5.3.

## 5. Conclusions

The mechanisms activated by plants to tolerate the presence of HMs were studied by exposing plants to not only a single HM but to a combination of Cd and Pb. Uptake and translocation strategies are not regulated by the same genes as suggested by the strong differences observed among genotypes in response to the two toxic HMs. In fact, the level of Cd in durum wheat roots and leaves is influenced by the co-presence of Pb and vice versa even if the phenomenon has different extent among genotypes.

Furthermore, nicotianamine and mucigenic acid seem to play a key role in response to Cd stress, probably by chelating the metal and avoiding its translocation to the plant shoots. The combined stress with Cd and Pb did not affect this mechanism, which appeared to be specific for Cd.

## Supplementary Materials

Supplementary Materials can be found at https://www.mdpi.com/1422-0067/20/23/5891/s1.
