# Supplementary material for: Combined Effect of Cadmium and Lead on Durum Wheat
Source: Int J Mol Sci. 2019 Nov 24;20(23):5891. doi: 10.3390/ijms20235891 (PMC6929116; doi:10.3390/ijms20235891)
Supplement: Supplementary file 1 [file ijms-20-05891-s001.pdf]

Table S1. Primers used for RT-PCR to measure expression levels of analysed genes.

Name	sequence 5'-3'	Tm (°C)
bHLH29_F	CCGTGCGGTCGCTGAT	59
bHLH29_R	CGCGTCGTGTCAACTCTCA	58
bHLH38_F	CGGACACAGTTGGAGCTATGC	59
bHLH38_R	CTGGCTAGTGCCAACTGCAA	59
bHLH47 _F	GCTACACTGTTCGTCTTCCATCCT	60
bHLH47 _R	TGGCGAGGTACCCAACACA	60
WRKY33_F	CCCACCGTTGTGCACTTGTA	59
WRKY33_R	GAGGAAGTACGGGCAGAAGGT	58
ZIP4_F	CAAGGCAACGTATGAGCACACT	59
ZIP4_R	TCCTGAGCCGGAGGATGAG	60
ZTP29_F	CACCGGGAAGTTTTGCAAA	58
ZTP29_R	TGAGACACCGAAGAAGAGTCATCT	58
YSL1_F	CTTGTTGCTGGCACCCTAGTC	58
YSL1_R	CCGTCTTGAAGTCTTGCATCAA	59
YSL2_F	CACATAGAATGTCAGCGGTGAGA	59
YSL2_R	CAATGCTTATCGCCCAGATCA	60
ZIF1_F	GGAGCAGGCGTTCTGTTCTC	59
ZIF1_R	ATCATTTGATCCCCTGGAAAGA	58
ZIFL1_F	TTCTCATGGGCGCAAAAAC	58
ZIFL1_R	AACCAGCATCAAGAATACCACTTG	58
ZIFL2_F	GCAGGAGCAGGTGTTCTGTTC	59
ZIFL2_R	TCATTTGATCCCCTGGAAAGA	58
HMA5_F	GCACCTTGAACTGACGCCTATT	59
HMA5_R	AATTACATATGCACACGAATCAATCA	58
NAS2_F	CTCCGCGTTGGCAAACTC	59
NAS2_R	GTGCAGGTTCGGCAAGATG	59
NAS3_F	TCATCTGTCGTGAACTCGTCATT	58
NAS3_R	GGCACAAACATCAGAACACACA	58
NAS4_F	TCAGCGGCACACATTTTCTC	58
NAS4_R	GGGCATCCATGTTATAGTTGCTT	58
NAAT_F	ATGGATTCCAGCGAGTCGAT	58
NAAT_R	GCTGGAGGTTCGACATTTCG	59
NADH_F	CCCCCTAGAGTAGCTGTTAATACGAA	60
NADH_R	GTTCAACTTTATGTATTCCTCTATCCGTAGA	60
